# Genomic signatures of positive selection in Awarik dromedary camels from southwestern of Saudi Arabia

**DOI:** 10.3389/fvets.2024.1443748

**Published:** 2024-09-18

**Authors:** Faisal Almathen

**Affiliations:** ^1^Department of Public Health, College of Veterinary Medicine, King Faisal University, Al-Hofuf, Saudi Arabia; ^2^Camel Research Center, King Faisal University, Al-Hofuf, Saudi Arabia

**Keywords:** dromedary, positive selection, evolutionary adaptation, haplotype-based statistics, camels

## Abstract

**Introduction:**

The Awarik camel population in southwestern Saudi Arabia exhibits unique genetic and phenotypic traits compared to other domestic camel populations. This study aims to explore the genomic signatures of positive selection in Awarik camels to understand their evolutionary history and identify genetic adaptations potentially shared with East African camel populations.

**Methods:**

Whole genome sequencing data from nine Awarik camels were analyzed using two robust intra-population haplotype-based statistical methods: integrated haplotype score (iHS) and number of segregating sites by length (nSL). These analyses were conducted to identify candidate regions under positive selection within the Awarik camel genome.

**Results and discussion:**

These analyses identified 66 and 53 candidate selection regions, encompassing 185 and 123 genes, respectively. The iHS analysis revealed significant selection signals on chromosomes 15 and 16, including a robust overlap on chromosome 15 (10 regions) involving the TRNAI-AAU gene, suggesting its critical role in adaptive processes. Additionally, chromosome 3 exhibited the highest number of candidate regions totaling 10. The nSL analysis highlighted statistically significant regions on chromosomes 2 and 7, as well as a high concentration of candidate regions on chromosome 14, totaling five regions. Notably, large candidate regions were also identified on chromosome 11 (200 kb: 51.750–51.950 kb) and chromosome 9 (325 kb: 45.825–46.150 kb). Functional annotation of these genes revealed involvement in diverse biological processes including olfactory activity, immune regulation, metabolism, insulin secretion, reproductive performance, kidney function, and cellular signaling, with specific genes like BAG5, septin 7, SLC13A1, PCED1B, BMPR1B, ZAR1, JAKMIP2, and NOTCH2 highlighted. These findings contribute to our understanding of the adaptive mechanisms of Awarik camels and have important implications for breeding and conservation strategies. Further research on these genetic adaptations, particularly those affecting immune response, is crucial to mitigate the impacts of climate change on camel populations.

## 1 Introduction

The Awarik camel population in Saudi Arabia is distinguished by its unique geographical distribution, phenotypic traits, and genetic characteristics. Studies have consistently demonstrated that the Awarik camels, particularly those from the western and southwestern regions, possess distinct genetic traits when compared to other camel populations, particularly those in the northern and central regions of the country (1). The uniqueness. The uniqueness of the Awarik camel population has been highlighted in various studies, indicating that these camels represent a genetically distinct group within Saudi Arabia (2–5). Recent research, including the study by Bahbahani et al. (6) which analyzed the whole genomes of 40 dromedary camels from across the Arabian Peninsula, has provided pivotal insights into the geographical genetic distinctions and regions under positive selection in dromedaries. This research has identified specific regions and haplotype blocks associated with adaptive physiological traits that are crucial for conservation. Building upon these comprehensive findings, this study focuses on exploring the unique genomic signatures of positive selection in the Awarik population. By examining these specific genetic adaptations, w aim to further understand the evolutionary pressures and adaptations that have uniquely shaped the Awarik camels, emphasizing the necessity for focused research on this distinct population.

The consistency of the Awarik camel population is evidenced by both genetic and demographic data. Genetically, the Awarik camels exhibit significant differentiation from other regional populations, reflecting a high degree of genetic homogeneity and stability within this group. This is supported by specific alleles and haplotypes that are consistent across generations, indicative of strong selective pressures and limited gene flow with other populations. Demographically, the population size has been relatively stable, as suggested by historical and recent surveys. This stability is crucial for preserving genetic integrity and ensures the reliability of evolutionary and adaptive studies focused on this unique group. Together, these genetic and demographic aspects confirm the Awarik population's consistency, underscoring its suitability for detailed genomic and adaptive analyses.

These one-humped dromedary camels are primarily located in the southwestern region of the country, which features a semi-arid climate with variable humidity levels, particularly high along the western coasts and mountains. Historically, Awarik camels share genetic ties with camel populations from the Horn of Africa, including Kenya, Somalia, and Sudan, regions that collectively host the largest camel populations in Africa.

Awarik camels have adapted to thrive in the harsh desert environment and humid conditions of the Red Sea coastal areas. Traditionally bred by local tribes for their milk and meat, these camels are highly valued for their resilience, endurance, and adaptability to coastal and mountainous terrains. They graze on the arak plant (*Salvadora persica*), which is abundant in their natural habitat. With unique physiological and behavioral traits, Awarik camels exhibit efficient temperature regulation and heat tolerance, enabling them to flourish in their coastal and mountainous environments (2).

Named after the arak plant, a significant part of their diet, the Awarik camel population is primarily found near the Red Sea coast of Saudi Arabia and is colloquially known as “Beach camels.” Predominantly concentrated in the Jazan region, these camels are characterized by a light brown coat, almost white, and short hair. They typically have a well-developed udder, medium neck circumference, pointed ears, and a hump positioned toward the hind of the back, giving them a shorter stature compared to other desert camels (7, 18). Awarik camels exhibit moderate milk production, with total lactation yields averaging 1,047.5 ± 11 liters (8).

This study aims to analyze the genomic signatures of positive selection in Awarik camels, seeking to uncover their evolutionary history and identify the genetic factors associated with their adaptation. Insights from this research can inform camel breeding programs, enhance conservation efforts, and develop strategies to mitigate the impact of climate change on camel populations.

## 2 Materials and methods

### 2.1 Sample collection and whole genome sequencing

Blood samples were obtained from nine unrelated female Awarik camels residing in the southwestern regions (Jazan) of Saudi Arabia, selected based on stringent phenotypic criteria. Genomic DNA was extracted from these samples using the Puregene^®^ Blood Core Kit C (Qiagen) according to the manufacturer's instructions. Subsequently, the extracted DNA underwent sequencing on the Illumina NovaSeq 6000 platform, employing a 150 bp paired-end approach. This sequencing was conducted at the Beijing Genomics Institute in China. The sequences data were deposited at European Nucleotide Archive Bioproject number: PRJEB47650.

### 2.2 Whole genome sequence read processing and variant calling

Whole genome sequences from the Awarik camels were aligned to the African dromedary reference genome (CamDro3) (9) using the *bwa-mem* algorithm of Burrows-Wheeler Aligner version 0.7.17 (10). Post alignment, reads were organized by coordinates using the *SortSam* and PCR duplicates were removed using the *MarkDuplicates* tool with (*REMOVE*_*DUPLICATES*=true) from Picard tools version 3.0.0 (http://broadinstitute.github.io/picard/index.html). SNPs calling was performed on Awarik autosomes using the *HaplotypeCaller* algorithm in GVCF mode of Genome Analysis Toolkit (GATK) version 4.2.5.0 (19). Autosomal SNPs were then filtered based on the criteria outlined by Bahbahani et al. (6), retaining those with a depth of coverage within ten reads and three standard deviations from the mean across all samples, for subsequent selection signature analyses.

### 2.3 Single nucleotide polymorphism quality control and pruning

A total of 5,720,174 autosomal SNPs were subjected to quality control pruning using PLINK v1.9 (11). SNPs were excluded based on the following criteria: a call rate below 100% of the genotyped samples, deviation from Hardy-Weinberg equilibrium *(P* < 1 × 10^−6^), or a minor allele frequency (MAF) of 5% or less. After filtering, 3,364,881 SNPs remained for selection signature analysis (Table 1). Samples were also evaluated and would be excluded for a genotyping call rate under 100% or a maximum pairwise identity-by-state (IBS) of 95% or greater; however, no samples met these exclusion criteria.

**Table 1:** Quality control criteria and the number of excluded and remaining SNPs for the signatures of selection analysis.

**Quality control criteria**	**Number of excluded SNPs**	**Remaining SNPs**
**Raw autosomal SNPs**	–	**5,720,174**
**Genotypic Call Rate** ** < 100%**	−81,638	5,638,536
**MAF** **≤5%**	−2,273,655	3,364,881
**HWE (*****P*****-value** **<** **1** **×10**^**−6**^**)**	0	3,364,881
**Final number of SNPs**	–	**3,364,881**

### 2.4 Signatures of selection analysis

Signatures of selection analyses were conducted on nine Awarik dromedary camels from southwestern Saudi Arabia using two intra-population haplotype-based statistics, integrated haplotype score (iHS) (12) and number of segregating sites by length (nSL) (13). These statistics were converted to *P*-values based on fractional ranks using the *stat*_*to*_*pvalue* based on the fractional ranks. These *P*-values were further transformed into rank-based values using two-tailed tests. Windows displaying–log10 (*P*-values) ≥ 4 (equivalent to *P* ≤ 0.0001) were defined as candidate windows with signatures of selection.

### 2.5 Functional annotation and enrichment analysis

The coordinates of the candidate regions were cross-referenced against the dromedary camel reference genome assembly CamDro3 gene list using the *GenomicRanges* package (14) in R. Functional profiling of the overlapping genes was conducted using the g: GOSt function of gProfiler (15), which identified functionally enriched terms for gene ontology biological processes and molecular functions. The gProfiler g: SCS algorithm was employed to compute multiple testing corrections for *P*-values from the gene ontology and pathway enrichment analyses. All identified genes were also analyzed using the functional annotation tool in the Database for Annotation, Visualization and Integrated Discovery (DAVID) Bioinformatics resource version 6.7 (16, 17) to determine enriched functional terms. An enrichment score of 1.3, equivalent to a Fisher exact test *P*-value of 0.05, was used as the threshold to define significantly enriched functional terms compared to the dromedary reference genome background. The genes were then cross-referenced with the literature to evaluate their relevance to the dromedaries' environmental adaptations and physiological traits.

## 3 Results

### 3.1 Summary statistics of the mapped sequence reads

The depth of coverage for the mapped sequence reads among the dromedary samples ranged from 19X to 22X, with an mean of 21X. On average, 99.7% of the sequence reads were mapped to the dromedary reference genome, and 95% of these were properly paired. The mapped reads covered ~94.7% of the reference genome.

### 3.2 Signatures of selection analysis of the two analysis

**Figure 1:**
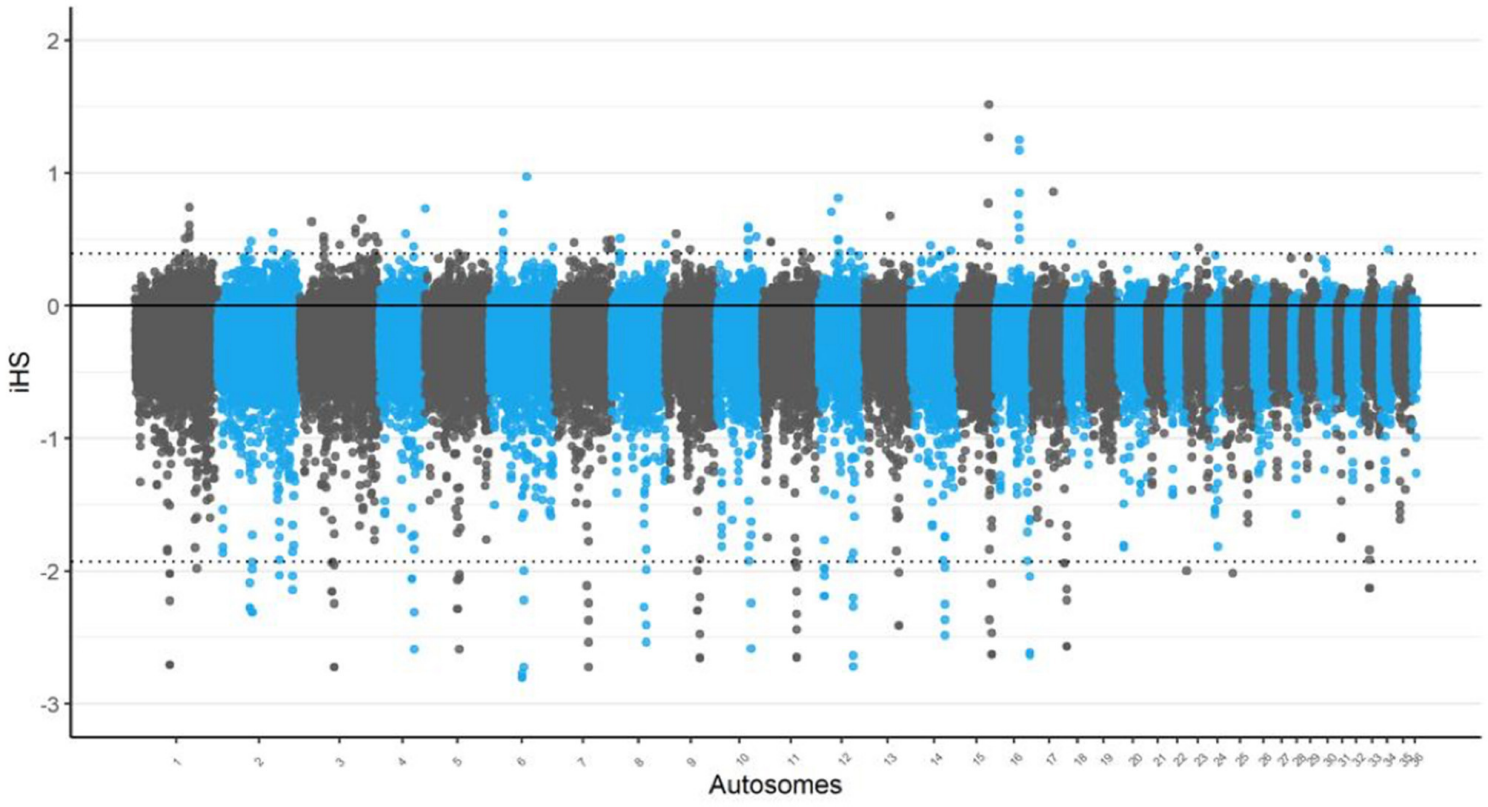
Signatures of selection analysis on autosomes of Awarik dromedary camels. Manhattan plots of genome-wide iHS analysis with a two-tailed Z-test applied and the significance threshold is set at–log10 (two-tailed *P*-value) = 4.

In the iHS analysis, the most significant regions were identified on chromosomes 15 and 16 as depicted in Figure 1. These regions displayed the strongest signals of selection, indicating that they may play a crucial role in the genetic adaptation of Awarik camels. Notably, a region on chromosome 15 was identified by both the iHS and nSL analyses, with the TRNAI-AAU gene located within this overlapping region, indicating a robust selection signal that may be significant for adaptive processes in camels. Additionally, chromosome 3 exhibited the highest number of candidate regions, with a total of 10 identified (see Supplementary Tables S1, S5).

**Figure 2:**
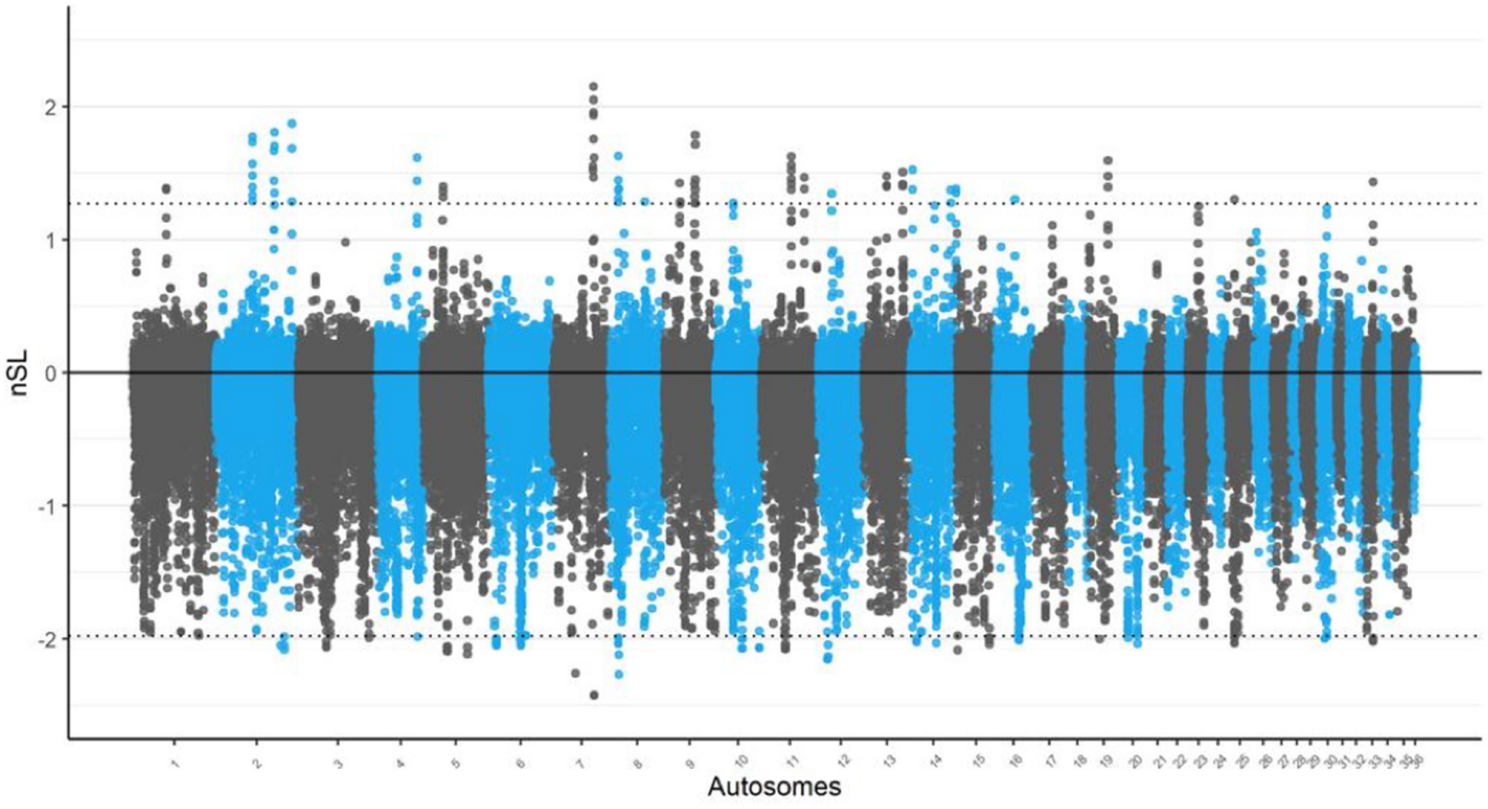
Signatures of selection analysis on autosomes of Awarik dromedary camels. Manhattan plots of genome-wide nSL analysis with a two-tailed Z-test applied and the significance threshold is set at–log10 (two-tailed *P*-value) = 4.

Conversely, the nSL analysis, chromosomes 2 and 7 exhibited the most statistically significant selection signals as shown in Figure 2. Although these regions were not the largest in terms of selection signal size, their high statistical significance makes them noteworthy. In particular, chromosome 2 showed a pronounced peak that warrants further investigation to identify potential candidate genes related to adaptive traits in Awarik camels. The nSL analysis also revealed a high concentration of candidate regions on chromosome 14, totaling five regions. Furthermore, the largest candidate regions were located on chromosome 11 (spanning 200 kb: 51.750–51.950 kb) and chromosome 9 (spanning 325 kb: 45.825–46.150 kb) in the iHS and nSL analyses, respectively (see Supplementary Tables S1, S5).

### 3.3 Functional annotation of candidate regions and enrichment analysis

#### 3.3.1 Integrated haplotype score (iHS)

The iHS analysis identified 185 genes within the 66 candidate selection regions (Supplementary Table S1). Functional profiling of these genes revealed several enriched molecular and biological processes, including the Wnt signaling pathway (see selected results in Table 1, Supplementary Tables S1, S2). However, none of these processes were significantly enriched. DAVID analysis identified six functional clusters, showing enrichment for functions related to olfactory activity (enrichment score = 4.65), immunoglobulin subtype (enrichment score = 0.65), ATP binding (enrichment score = 0.41), basic and acidic residues (enrichment score = 0.24), and zinc finger C2H2-type/integrase DNA-binding domain (enrichment score = 0.13). Literature review highlighted candidate genes involved in immune regulation and inflammatory responses, metabolism, cell signaling and receptor regulation, neuronal development and function, and cytoskeleton and cellular structure (Table 2).

**Table 2:** Biological functions of candidate genes under selection using iHS analysis on Awarik dromedary camel autosomes.

**Functional category**	**Gene ID**	**Candidate region (chromosome: start-stop)**
Immune regulation and inflammatory responses	BAG5	6: 95.53–95.54 Mb
	septin 7	7: 48.49–48.58 Mb
	N4BP1	9: 51.77–51.86 Mb
	DOCK9	14: 60.02–60.28 Mb
	HTR7	11: 51.89–51.97 Mb
Translation and protein synthesis	TRNAT-GGU	4: 58.64–58.64 Mb
	TRNAI-AAU	15: 48.61–48.61 Mb
	TRNAT-UGU	16: 35.97–35.97 Mb
	EIF3F	10: 39.45–39.46 Mb
Cell/receptors signaling and regulation	BMPR1B	2: 55.21–55.56 Mb
	JAKMIP2	3: 10.73–10.74 Mb
	NOTCH2	9: 23.18–23.33 Mb
	GNAO1	9: 45.71–45.88 Mb
	WARS2	9: 22.64–22.73 Mb
	CES5A	9: 45.88–46.11 Mb
	CES1	9: 46.13–46.16 Mb
	ATOH7	11: 35.45–35.48 Mb
	YAP1	10: 65.71–65.82 Mb
	CRK	16: 35.70–35.73 Mb
	CDK8	14: 34.98–35.83 Mb
	MYO1C	16: 35.73–35.76 Mb
	WASF3	14: 36.64–37.41 Mb
	YWHAE	16: 35.65–35.69 Mb
	INPP5K	16: 35.76–35.78 Mb
	TMX4	19: 28.99–29.10 Mb
	PLCB1	19: 29.14–29.81 Mb
	RIMS2	25: 11.59–12.10 Mb
	DPYS	25: 12.21–12.29 Mb
	TMPRSS13	33: 12.25–12.28 Mb
	5-hydroxytryptamine receptor 3C-like	1: 51.80–51.81 Mb
	5-hydroxytryptamine receptor 3E	1: 51.84–51.85 Mb
	CASC4	6: 20.59–20.69 Mb
	PTPRN2	7: 85.74–86.12 Mb
	NT5DC3	12: 28.86–28.92 Mb
	5-hydroxytryptamine receptor 3C-like	1: 51.82–51.82 Mb
Metabolism	SLC13A1	7: 60.76–60.83 Mb
	PCED1B	12: 14.12–14.26 Mb
	CLYBL	14: 60.65–60.86 Mb
	PIGB	6: 11.95–11.98 Mb
	PIGBOS1	6: 11.98–11.98 Mb
	FXYD6	33: 12.30–12.33 Mb
	FXYD2	33: 12.34–12.35 Mb
	Creatine kinase (CKB)	6: 95.50–95.51 Mb
	ER-resident protein 44 (ERp44)	4: 39.21–39.28 Mb
	GLT8D2	12: 28.71–28.75 Mb
	Wdr78	13: 51.72–51.80 Mb
Cellular/membrane transport and vesicle trafficking	SLC10A4	2: 87.91–87.91 Mb
	SLC2A13	12: 19.47–19.83 Mb
	RAB27A	4: 58.58–58.60 Mb
	DOC2B	16: 35.61–35.64 Mb
Cytoskeleton and cellular structure	SLAIN2	2: 87.95–88.02 Mb
	RBMS1	5: 35.33–35.53 Mb
	TBX15	9: 22.50–22.61 Mb
	ST6GALNAC3	13: 58.80–59.34 Mb
Protein synthesis and modification	EIF2B5	1: 51.86–51.87 Mb
	RPL24	1: 81.72–81.72 Mb
	60S ribosomal protein L17 pseudogene	2: 48.65–48.66 Mb
	60S ribosomal protein L34 pseudogene	3: 94.63–94.64 Mb
	MRPS27	3: 48.51–48.61 Mb
	PTCD2	3: 48.61–48.64 Mb
	SENP7	1: 81.79–81.92 Mb
	UBE2E3	5: 51.84–51.92 Mb
Enzymes and catalytic activity	ALG3	1: 51.94–51.95 Mb
	ACSL6	3: 94.68–94.74 Mb
	Dram2	9: 16.02–16.04 Mb
	CTDSPL2	6: 20.52–20.59 Mb
	PRKG1	11: 47.54–48.65 Mb
	MSH2	15: 34.92–34.99 Mb
Transporters and Ion channels	SLC39A8	2: 50.04–50.11 Mb
	SLC35D1	13: 51.87–51.92 Mb
	ZFP62	3: 11.76–11.76 Mb
Nuclear and transcriptional regulation	TAF1D	10: 60.09–60.10 Mb
	NR2C2	17: 48.56–48.64 Mb
	UROC1	17: 51.78–51.81 Mb
	ZXDC	17: 51.81–51.84 Mb
	Tdg	12: 28.73–28.77 Mb
Cellular structure and motility	LAMTOR3	2: 51.83–51.85 Mb
	DAPP1	2: 51.85–51.90 Mb
	ESyts 2	7: 86.33–86.40 Mb
	WDR60	7: 86.42–86.47 Mb
Neuronal development and function	ASTN2	4: 51.26–52.06 Mb
	EpCAM	15: 34.90–34.92 Mb
	CNTNAP2	7: 77.81–79.82 Mb
	VSTM5	10: 60.15–60.17 Mb
	Cntn1	12: 18.76–19.05 Mb

Functional categories encompass examples of candidate genes and their respective candidate regions.

#### 3.3.2 Number of segregating sites by length (nSL)

The nSL analysis identified 123 genes within the 53 candidate selection regions (Supplementary Tables S4, S5). Functional profiling of these genes revealed several enriched molecular and biological processes, including the Wnt signaling pathway (see selected results in Table 2, Supplementary Tables S4, S5). However, none of these processes were significantly enriched. DAVID analysis identified five functional clusters, showing enrichment for functions related to transmembrane helices (enrichment score = 0.52), olfactory and transducer activity (enrichment score = 0.39), immunoglobulin-like domains (enrichment score = 0.38), cell and plasma membranes (enrichment score = 0.38), and ion and zinc binding (enrichment score = 0.20). Literature review highlighted several candidate genes associated with key biological processes such as reproductive performance, immune response, neuroplasticity, insulin secretion and signaling, as well as kidney absorption and reabsorption (Table 3, Supplementary Table S6). These genes are of particular interest due to their roles in physiological adaptations that are critical for the survival and reproductive success of Awarik camels in challenging environments.

**Table 3:** Biological functions of candidate genes under selection using nSL analysis on Awarik dromedary camel autosomes.

**Functional category**	**Gene ID**	**Candidate region (chromosome: start-stop)**
Immune response	JAKMIP2	03: 10.73–10.74 Mb
	NOTCH2	09: 23.18–23.33 Mb
Metabolism/enzymes	PBLD	11: 35.41–35.45 Mb
	CDK8	14: 34.98–35.83 Mb
	INPP5K	16: 35.76–35.78 Mb
	PITPNA	16: 35.78–35.81 Mb
	CLYBL	14: 60.65–60.86 Mb
	DPYS	25: 12.21–12.29 Mb
	TMPRSS13	33: 12.25–12.28 Mb
	FXYD6	33: 12.30–12.33 Mb
	INPP5K	16: 35.76–35.78 Mb
	PITPNA	16: 35.78–35.81 Mb
	TLCD2	16: 35.92–35.92 MB
Insulin secretion and signaling	DOC2B (105088125)	16: 35.61– 35.64 MB
	MYO1C (105088122)	16: 35.73– 35.78 MB
Cellular components/ structural genes	RUFY2	11: 35.36–35.40 Mb
	PBLD	11: 35.41–35.45 Mb
	PCED1B	12: 14.12–14.26 Mb
	AMIGO2	12: 14.26–14.26 Mb
	ZSWIM5	13: 34.00–34.13 Mb
	ST6GALNAC3	13: 58.80–59.34 Mb
	CRK	16: 35.70–35.73 Mb
	MYO1C	16: 35.73–35.76 Mb
	WDR81	16: 35.93–35.94 Mb
DNA/RNA binding/ transcription/ translation	EIF3F	10: 39.45–39.46 Mb
	HNRNPH3	11: 35.40–35.41 Mb
	TRNAI-AAU	15: 48.61–48.61 Mb
	DOC2B	16: 35.61–35.64 Mb
	RPA1	16: 36.00–36.05 Mb
	TMX4	19: 28.99–29.10 Mb
Signal transduction/ cell communication	SLC13A1	07: 60.76–60.83 MB
	TUB	10: 39.35–39.43 Mb
	YAP1	10: 65.71–65.82 Mb
	YWHAE	16: 35.65–35.69 Mb
	MYO1C	16: 35.73–35.76 Mb
	CRK	16: 35.70–35.73 Mb
Membrane transport	SLC2A13	12: 19.47–19.83 Mb
	WASF3	14: 36.64–37.41 Mb
	PITPNA	16: 35.78–35.81 Mb
Cell cycle/ development	ATOH7	11: 35.45–35.48 Mb
	MYPN	11: 35.48–35.57 Mb
	WDR81	16: 35.93–35.94 Mb
	DSCAML1	33: 12.38–12.68 Mb

Functional categories encompass examples of candidate genes and their respective candidate regions.

## 4 Discussion

This study provides a comprehensive genomic analysis the Awarik camel population, indigenous to the southwestern region of Saudi Arabia, offering significant insights into the genetic mechanisms underlying their adaptation to challenging environments (2, 3). Through high-depth whole genome sequencing, we identified key genomic regions under positive selection, shedding light on the evolutionary strategies that have enabled these camels to thrive in semi-arid and coastal habitats (6). The identification of significant selection regions on chromosomes 2 and 7 in the nSL analysis, and on chromosomes 15 and 16 in the iHS analysis, emphasizes the complexity of the selection landscape in Awarik camels. The statistically significant signals on chromosomes 2 and 7 suggest that these regions may harbor genes of adaptive significance, potentially influencing traits that are critical for survival in the harsh environments inhabited by these camels. In particular, the overlap identified on chromosome 15, where the TRNAI-AAU gene was detected in both the iHS and nSL analyses, strengthens the case for importance of this gene. which likely plays a pivotal role in the genetic adaptation of Awarik camels. Further functional analysis is needed to elucidate its specific role in camel physiology and adaptation.

Our analysis highlighted significant selection signatures on chromosomes 3, 14, 11, and 9, each pointing to different aspects of genetic adaptation. Chromosomes 3 and 14, which showed the highest number of candidate regions in the iHS and nSL analyses, respectively, are particularly important as they likely host genes conferring adaptive advantages in response to the specific selection pressures faced by the Awarik population (Supplementary Tables S1, S5). This pattern suggests a tailored response to the unique selection pressures faced by the Awarik population, potentially reflecting localized adaptations not as pronounced in other regional camel populations.

Moreover, the notable presence of large candidate regions on chromosomes 9 and 11 underscores the importance of these loci in the adaptation of the Awarik camels. Previous studies such as those by Bahbahani et al. (6) and Al Abri et al. (5) have reported similar high-frequency haplotypes on these chromosomes in mixed groups of dromedaries, including Awarik camels. The consistency of these findings across studies supports the hypothesis that these genomic regions may hold unique significance for the Awarik population, necessitating further research to fully understand their biological relevance.

Functional annotation of these regions revealed involvement in diverse biological processes critical to survival in extreme environments, such as immune regulation, metabolism, and reproductive performance. Although no significant enrichment was found in pathways like Wnt signaling, the recurring identification of genes associated with this pathway across different analyses suggest its potential role in developmental and adaptive processes (15). The DAVID analysis further supported this by identifying functional clusters related to sensory functions, immune responses, and metabolic processes, all of which are vital for thriving in the variable climates of the Red Sea coastal areas (16, 17). These findings imply a complex and multifaceted genetic basis for the adaptation Awarik camel, involving various physiological and cellular processes.

The genes uncovered in this study, particularly those involved in olfactory activity, immune response, and kidney function, highlight the Awarik camel's genetic specialization for their diet and environmental stressors. These adaptations appear to be deeply embedded in the genetic fabric of the population, suggesting a complex evolutionary history that has finely tuned these animals to their specific ecological niches (5).

Furthermore, the implications of these genomic insights extend beyond academic understanding to practical applications in breeding and conservation. The detailed genetic markers identified here can guide selective breeding programs aimed at enhancing desirable traits like resilience to climate variability and disease resistance. Additionally, the genetic diversity revealed through this study underscores the importance of conservation strategies that preserve these unique genetic resources, which are invaluable for the Awarik camel's continued adaptation and survival (1).

Future research should build on on these findings by increasing the sample sizes and incorporating comparative genomic studies with other camel populations, including those from East Africa with whom the Awarik camels share historical ties (9). Such studies could illuminate both shared and unique adaptations, providing a broader understanding of camel evolution across different environments. Integrating environmental and phenotypic data could refine the connections between genetic adaptations and specific environmental challenges, enhancing the predictive power of genomic studies in conservation and management practices.

In conclusion, the genomic signatures of positive selection identified in the Awarik camels not only deepen our understanding of their unique adaptations but also provide a foundational knowledge base for developing targeted interventions in breeding and conservation. These interventions are critical for sustaining the Awarik camel population amid the escalating pressures of climate change and habitat loss, ensuring their resilience and productivity for future generations.

## Data Availability

The data presented in the study are deposited at European Nucleotide Archive Bioproject number: PRJEB47650.
